# Pre-operative levels of angiopoietin protein-like 3 (ANGPTL3) in women diagnosed with high-grade serous carcinoma of the ovary

**DOI:** 10.1186/s12944-024-02038-8

**Published:** 2024-02-27

**Authors:** Emilie Wong Chong, France-Hélène Joncas, Pierre Douville, Dimcho Bachvarov, Caroline Diorio, Frédéric Calon, Ann-Charlotte Bergeron, Jonatan Blais, Shuk On Annie Leung, Nabil Georges Seidah, Anne Gangloff

**Affiliations:** 1 Faculty of Medicine, Laval University, Québec, QC Canada; 2 Centre de recherche sur le cancer (CRC) de l’Université Laval, Québec, QC Canada; 3 Réseau de Recherche sur le Cancer, 9 McMahon, Québec, QC G1R 3S3 Canada; 4 Oncology Research Division, CHU de Québec- Université Laval, Québec, QC Canada; 5 Centre des Maladies du Sein Deschênes-Fabia, Hôpital du Saint-Sacrement, Québec, QC Canada; 6 Faculty of Pharmacy, Laval University, Québec, QC Canada; 7 Neuroscience Research Division, CHU de Québec- Université Laval, Québec, QC Canada; 8 CHU de Québec-Université Laval, Lipid Clinic, Room C-00102, 2705 Laurier Blvd, Québec, QC G1V 4G2 Canada; 9 Division of Gynecologic Oncology, Department of Obstetrics and Gynecology, McGill University Health Center, Montreal, QC Canada; 10 Laboratory of Biochemical Neuroendocrinology, Institut de Recherches Cliniques de Montréal, Montreal, QC Canada

**Keywords:** ANGPTL3, Evinacumab, PCSK9, Apo CIII, Lp(a), Lipid profile, High-grade serous ovarian cancer

## Abstract

Cancer cells need constant supplies of lipids to survive and grow. Lipid dependence has been observed in various types of cancer, including high-grade serous ovarian carcinomas (HGSOC), which is a lethal form of gynecological malignancy. ANGPTL3, PCSK9, and Apo CIII are pivotal lipid-modulating factors, and therapeutic antibodies have been developed against each one (Evinacumab, Evolocumab and Volanesorsen, respectively). The roles -if any- of ANGPTL3, PCSK9, and Apo CIII in HGSOC are unclear. Moreover, levels of these lipid-modulating factors have never been reported before in HGSOC. In this study, circulating levels of ANGPTL3, PCSK9, and Apo CIII, along with lipid profiles, are examined to verify whether one or many of these lipid-regulating factors are associated with HGSOC.

**Methods **ELISA kits were used to measure ANGPTL3, PCSK9 and Apo CIII levels in plasma samples from 31 women with HGSOC and 40 women with benign ovarian lesions (BOL) before treatment and surgery. A Roche Modular analytical platform measured lipid panels, Apo B and Lp(a) levels.

**Results **ANGPTL3 levels were higher in women with HGSOC (84 ng/mL, SD: 29 ng/mL, *n* = 31) than in women with BOL (67 ng/mL, SD: 31 ng/mL, *n* = 40; HGSOC vs. BOL *P* = 0.019). Associations between the lipid panel and ANGPTL3, and the inverse relationship between HDL-cholesterol and triglycerides, were present in women with BOL but not with HGSOC. PCSK9 and Apo CIII were not associated with HGSOC.

**Conclusions **In this cohort of 71 women, ANGPTL3 levels were increased in HGSOC patients. The presence of HGSOC disrupted the classic inverse relationship between HDL and triglycerides, as well as the association between the lipid panel and ANGPTL3. These associations were only maintained in cancer-free women. Given the availability of Evinacumab, a therapeutic antibody against ANGPTL3, the current finding prompts an assessment of whether ANGPTL3 inhibition has therapeutic potential in HGSOC.

**Supplementary Information:**

The online version contains supplementary material available at 10.1186/s12944-024-02038-8.

## Background

Ovarian cancer (OC) is an aggressive form of cancer, with fewer than half of women who will survive beyond the 5 years following their diagnosis [1]. There is an urgent need for new approaches to treat women diagnosed with OC. In addition to the actual standard of care combining surgery and chemotherapy, cholesterol-lowering drugs appear as promising add-on therapeutics to impede ovarian cancer progression [2–4].

Statins are inhibitors of hydroxymethylglutaryl-coenzyme A reductase, the rate-limiting step in cholesterol de novo synthesis, and have been extensively investigated for their protective role against cancers [5–7]. Several studies indicate decreased OC-related mortality in women using statins, especially lipophilic ones [8–11]. Due to a lack of prospective randomized trials, no consensus has been reached on the benefits of using statins as an adjuvant treatment [12–16].

The effects of non-statin cholesterol-lowering drugs on OC progression are scarcely documented. Apolipoprotein C-III (Apo CIII), angiopoietin protein-like 3 (ANGPTL3), proprotein convertase subtilisin/kexin type 9 (PCSK9) modify circulating lipid levels (Low-density Lipoprotein or LDL, and high-density lipoproteins or HDL levels) and thus modulate lipid supplies to extra-hepatic tissues, including cancer cells. Lipoprotein (a), or Lp(a), is an LDL particle with an Apo (a) and is gaining traction in the cardiovascular field, while little is known about its role in cancers. These hepatic-derived factors are the targets of a new generation of cholesterol-lowering drugs. Volanesorsen is an antisense oligonucleotide which targets Apo CIII and was developed in hyperchylomicronemia [17]. Evinacumab is a monoclonal antibody (mab) that inhibits ANGPTL3 and is approved for treating homozygous hypercholesterolemia [18]. Evolocumab, also a mab, blocks PCSK9 and is combined with a statin to reach Apo B targets (< 0,70 g/L) in patients with cardiovascular diseases [19].

The present study aims to measure and report circulating levels of ANGPTL3, PCSK9 and Apo CIII in women with ovarian carcinoma (OC); all three lipid-modulating factors are the target of clinically available therapeutical monoclonal antibodies or antisense oligonucleotide therapy. The central hypothesis of this article is that at least one of these factors will be modified in OC to meet the increased cholesterol requirements of OC.

The present study reports measurements of circulating levels of Lp(a), Apo CIII, ANGPTL3, and PCSK9 in women diagnosed with either an epithelial high-grade serous ovarian carcinoma (HGSOC) or a benign ovarian lesion (BOL). Additionally, correlations linking these lipid-related factors to the lipid profile and ovarian tumor biomarkers CA125 and HE4 are provided. Furthermore, specific patterns related to women with HGSOC are outlined. This study is the first to simultaneously measure all the parameters described above and compare their associations across cancer-free and cancer patients from a same cohort.

## Patients and methods

### Patients

Participants included in the present study were sampled prior to treatment, of female biological sex, aged between 39 and 83 years old (y.o.), and had not yet undergone surgical debulking. Forty participants were diagnosed with a BOL (benign ovarian cyst-like lesion: serous para-tubular adenofibromas or cystadenofibromas). Thirty-one participants had received an epithelial high-grade serous ovarian carcinoma (HGSOC) diagnosis. All diagnoses were performed at the Pathology Department of the CHU de Québec-Université Laval. Ovarian cancer grading was determined using biomarker levels and histological analyses of biopsies. Staging of ovarian tumors was established according to the *Fédération Internationale de Gynécologie et d’Obstétrique* (FIGO) system. Mean age and median age were similar between the two groups (Table 1).

**Table 1:** Biological and biochemical characteristics of women diagnosed with benign ovarian lesion (BOL), high-grade serous ovarian carcinoma (HGSOC) and entire cohort (Total)

	Women with BOL (*n* = 40)	Women with HGSOC (*n* = 31)	Total	*P*-value (unpaired)	*P*-value (paired)
AGE (years)					
Mean (SD)	63.7 (9.4)	64.8 (9.6)	64.2 (9.5)		
Median [IQR]	64.7 [57.7; 70.9]	64.8 [58.3; 70.7]	64.8 [58.0; 71.0]	0.65 ^a^	0.13 ^d^
POST-MENOPAUSAL STATUS					
Yes (%)	33 (80.4)	21 (91.3)	54 (76.1)		
No (%)	4 (9.8)	2 (6.7)	6 (8.4)	0.29 ^b^	
Missing (%)	4 (9.8)	7 (23.3)	11 (15.5)		
TUMOR STAGE					
I	–	3	3		
II	–	3	3		
III	–	21	21		
IV	–	4	4		
TUMOR MARKERS					
CA125 (U/mL)					
Mean (SD)	21 (12)	422 (1358)	196 (913)		
Median [IQR]	17 [12; 27]	54 [29; 301]	27 [14; 53]	**1.4 E-7** ^c^****	**5.3 E-6** ^e^****
HE4 (pmol/L)					
Mean (SD)	66 (25)	539 (1185)	244 (754)		
Median [IQR]	57 [49, 83]	155 [93; 447]	77 [54; 129]	**2.2 E-7** ^c^****	**2.0 E-5** ^e^****
Missing	2 (5.0%)	8 (25.8%)	10 (14.1%)		
LIPIDS					
TG (mmol/L)					
Mean (SD)	1.45 (0.71)	1.46 (0.65)	1.45 (0.68)		
Median [IQR]	1.23 [0.95; 1.74]	1.34 [1.02; 1.63]	1.34 [0.99; 1.70]	0.87 ^c^	0.92 ^d^
Lp(a) (nmol/L)					
Mean (SD)	73.9 (88.0)	74.4 (98.0)	74.1 (91.8)		
Median [IQR]	34.5 [12; 106]	21.0 [7; 134]	28.0 [11; 111]	0.66 ^c^	0.63 ^d^
HDL (mmol/L)					
Mean (SD)	1.55 (0.46)	1.45 (0.33)	1.51 (0.41)		
Median [IQR]	1.48 [1.22; 1.76]	1.49 [1.15; 1.68]	1.49 [1.19; 1.73]	0.26 ^a^	0.24 ^d^
TC (mmol/L)					
Mean (SD)	5.35 (1.09)	5.39 (1.05)	5.37 (1.07)		
Median [IQR]	5.42 [4.41; 6.15]	5.16 [4.44; 6.26]	5.28 [4.43; 6.24]	0.93 ^c^	0.96 ^d^
Apo B (g/L)					
Mean (SD)	1.00 (0.23)	1.03 (0.26)	1.01 (0.24)		
Median [IQR]	0.99 [0.81; 1.16]	0.99 [0.85; 1.17]	0.99 [0.83; 1.16]	0.63 ^a^	0.82 ^d^
Non-HDL (mmol/L)					
Mean (SD)	3.79 (1.00)	3.94 (1.02)	3.86 (1.01)		
Median [IQR]	3.73 [2.91; 4.56]	3.77 [3.08; 4.76]	3.77 [3.01; 4.70]	0.55 ^a^	0.72 ^d^
LDL (mmol/L)					
Mean (SD)	3.13 (0.98)	3.28 (0.97)	3.20 (0.98)		
Median [IQR]	3.08 [2.38; 3.80]	3.17 [2.39; 3.98]	3.10 [2.38; 3.84]	0.54 ^a^	0.69 ^d^
LIPID-RELATED FACTORS					
Apo CIII (μg/mL)					
Mean (SD)	526 (241)	615 (306)	569 (276)		
Median [IQR]	485 [404; 616]	562 [459; 826]	508 [404; 688]	0.15 ^c^	0.33 ^d^
Missing	8 (20.0%)	1 (3.2%)	9 (12.7%)		
ANGPTL3 (ng/mL)					
Mean (SD)	67 (31)	84 (29)	74 (31)		
Median [IQR]	65 [46; 86]	77 [66; 102]	70 [51; 92]	**0.019** ^a^ *	**0.030** ^d^ *
PCSK9 (ng/mL)					
Mean (SD)	108 (32)	103 (38)	106 (35)		
Median [IQR]	104 [87; 118]	97 [79; 113]	103 [82; 117]	0.33 ^c^	0.44 ^d^

*TG* triglycerides, *Lp(a)* lipoprotein (a), *TC* total cholesterol, *Apo B* apolipoprotein B

^a^ Student T-test, ^b^ Fisher’s Exact Test for count data, ^c^ Wilcoxon Mann Whitney U test, ^d^ Paired Student T-test, ^e^ Paired Wilcoxon signed rank test

Boldface indicates significance with *: *P* < 0.05, ****: *P* < 0.000 1

### Blood samples

Plasma specimens from 71 women newly diagnosed with HGSOC or BOL were collected between 2017 and 2020 and were registered in the biobank of The Cancer Research Network (*RRCancer, Montréal, QC)*. Plasma samples collected in EDTA vacutainers were centrifuged, aliquoted into 500 μL fractions and then stored at − 80 °C. Aliquots were thawed only once, on the day of measurements. All clinical samples were anonymized before analysis to protect patients’ privacy. All methods described below were carried out in accordance with relevant ethical guidelines and regulations.

### Lipid profile

Three-hundred μL of each sample were sent to the Core laboratory of the *Hôpital de l’Enfant-Jésus* (Quebec City, Canada) for measurements of apolipoprotein B (Apo B, in g/L), Lp(a) (nmol/L), total cholesterol (TC, in mmol/L), HDL-cholesterol (HDL, in mmol/L) and triglycerides (TG, in mmol/L) on a Cobas 8000 Modular analytical platform (Roche Diagnostics). Lp(a) measurements lower than the reportable range (Lp(a) < 10 nmol/L) were set to a default value of 3 nmol/L, corresponding to half of the lower limit of detection. LDL-cholesterol (LDL) was calculated with the Friedewald equation: LDL in mmol/L = TC – (TG/ 2.2) – HDL [20]. Non-HDL cholesterol was calculated by subtracting HDL-cholesterol from total cholesterol in mmol/L.

### PCSK9, ANGPTL3 and Apo CIII

Using commercially available Enzyme-Linked Immunosorbent Assay (ELISA) kits, levels of ANGPTL3 (cat # ab254510, Abcam, Cambridge, MA, USA), PCSK9 (cat # 443107, Biolegend, San Diego, CA, USA) and Apo CIII (cat # ab154131, Abcam, Cambridge, MA, USA) were measured in 200 μL aliquots of plasma samples, in compliance with manufacturer’s instructions. All plasma samples were assayed in duplicate. Intra-assay (mean ± SD) & inter-assay coefficients of variation were: 4.0 ± 4.1% & 17.3% for ANGPTL3, 2.2 ± 1.9% & 15.1% for PCSK9 and 12.0 ± 10.7% & 23.4% for Apo CIII, respectively.

### Tumor markers

Levels of tumor markers were tested at the time of specimen collection and were retrieved from the biobank database: measurements of Carbohydrate Antigen 125 (CA125) and Human Epididymis protein 4 (HE4) have been described elsewhere [21].

### Statistical analysis

The minimal sample size for a statistical power of 80% and an alpha error of 0.05 was estimated using G*power software version 3.1. Forty women with a BOL and 31 women with a HGSOC formed the two study groups. BOL and HGSOC participants were age-matched (maximal difference of ±3 years) prior to paired-group analyses to limit the confounding effect of age on both cancer outcomes and blood lipid profiles. Two-group comparisons involved two-tailed tests admitting a type I error α = 0.05, performed using SAS software version 9.4 and R software version 4.2.0. Correlation coefficients between all variables were determined by Spearman’s rank order test, computed with the *corx* package (courtesy of Dr. James Conigrave, https://github.com/conig/corx). A small proportion of the analytes (13% for Apo CIII and 14% for HE4) were not successfully measured. Consequently, the number of subjects may vary depending on the applied statistical model. A flow chart of the analyses performed with adjustment for sparse data is provided in supplemental Fig. S1.

## Results

### Biological characteristics of the cohort

Participants’ age, menopausal status, tumor staging, tumor markers levels, lipid panel and lipid-related factors levels are listed in Table 1. Mean age was 64.2 ± 9.5 y.o. for the entire cohort and did not differ between women diagnosed with BOL vs women diagnosed with HGSOC (BOL: 63.7 ± 9.4 y.o., HGSOC: 64.8 ± 9.6 y.o., *P*-value BOL vs HGSOC =0.13). The majority of women were in postmenopausal status (80.4 and 91.3% for the BOL and HGSOC groups respectively). Menopausal statuses distribution was homogenous between groups (*P*-value of 0.29 with Fisher’s Exact Test).

### Lipid-related factors and malignancy

**Fig. 1:**
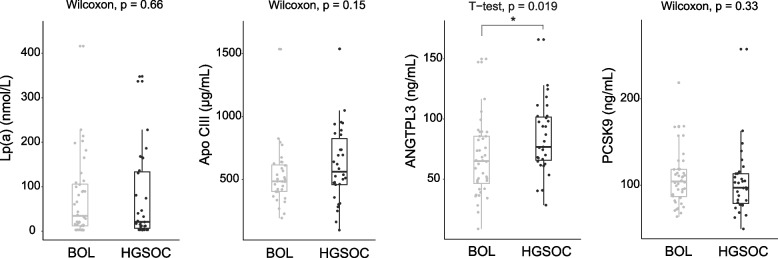
Plasma levels of Lp(a), Apo CIII, ANGPTL3, and PCSK9 in women diagnosed with benign ovarian lesion (BOL) and high-grade serous ovarian carcinoma (HGSOC). Significantly higher levels (*P* = 0.019) of ANGPTL3 were observed in HGSOC (*n* = 31) compared to BOL (*n* = 40) with T-test. *: *P* < 0.05

Plasma ANGPTL3 levels were higher in women with HGSOC compared to women diagnosed with a BOL (84 ± 29 ng/mL vs. 67 ± 31 ng/mL, *P* = 0.019; Table 1 and Fig. 1). The observed association remained when comparing age-matched BOL and HGSOC women (*n* = 31 per group, *P* = 0.030; Table 1). The presence of cancer did not modify Apo CIII and PCSK9 levels (see HGSOC and BOL, Table 1 and Fig. 1).

### Lipid profile

**Fig. 2:**
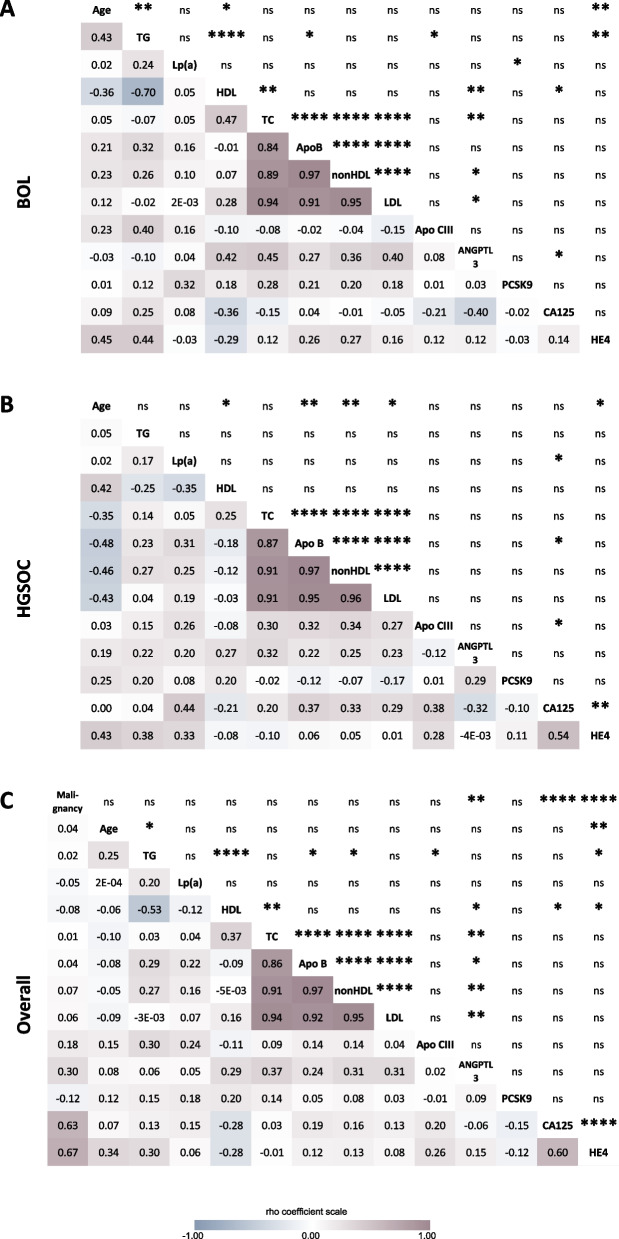
Pairwise Spearman’s correlation analyses between all variables. Pairwise correlations are presented for (**A**) benign ovarian lesion (BOL), (**B**) high-grade serous ovarian carcinoma (HGSOC) and (**C**) for the entire cohort (overall). In each matrix, correlation coefficients (rho) are displayed in the lower half-panel with color hues indicative of relationship strength (see rho coefficient scale) while significance is indicated in the upper half-panel (ns: non-significant, *: *P* < 0.05; **: *P* < 0.01; ***: *P* < 0.001; ****: *P* < 0.000 1)

Lipid profiles (TC or total cholesterol, HDL or High-density Lipoprotein cholesterol, TG or triglycerides, calculated LDL, calculated non-HDL, Apo B and Lp(a)) were similar between BOL and HGSOC groups (Table 1, all *P*-values > 0.05), with lipid levels comprised within the normal range [22]. A tight correlation between variables reflecting atherogenic particles (Apo B, non-HDL and LDL) was observed regardless of the group (all rho above 0.80, all *P*-values < 0.0001, Fig. 2). The classic inverse correlation between HDL and TG was seen in BOL (rho = − 0.70, *P*-value < 0.0001, Fig. 2A) and the entire cohort (rho = − 0.53, *P*-value < 0.0001, Fig. 2C). However, this correlation was not obtained (weak and non-significant) in HGSOC (rho = − 0.25, *P*-value > 0.05, Fig. 2B).

### Lipid-related factors and the lipid profile

A significant correlation between ANGPTL3 and several lipid parameters (HDL, total cholesterol, non-HDL cholesterol and LDL) was observed in BOL (Fig. 2A: HDL rho = 0.42, *P*-value < 0.01; TC rho = 0.45, *P*-value < 0.01; non-HDL rho = 0.36, *P*-value < 0.05; LDL rho = 0.40, *P*-value < 0.05) and in the entire cohort (Fig. 2C: HDL rho = 0.29, *P*-value < 0.05; TC rho = 0.37, *P*-value < 0.01; non-HDL and LDL both rho = 0.31, *P*-value < 0.01). No correlation between ANGPTL3 and lipid parameters were obtained in the HGSOC group (Fig. 2B). Similar correlations were obtained after age adjustment (Supplemental Fig. S2).

Apo CIII or PCSK9 levels were not correlated with the lipid profile in either group (Fig. 2). Nevertheless, Apo CIII levels correlated with TG levels in BOL and the entire cohort (Fig. 2A and C) but not in HGSOC (Fig. 2B). A weak correlation between PCSK9 and Lp(a) in BOL was also noticed (Fig. 2A).

### Ovarian cancer tumor markers

CA125 and HE4 are established tumor markers of OCs and, as such, displayed significantly higher levels in HGSOC than in BOL (Table 1 and Supplemental Fig. S3). CA125 increased in HGSOC (422 ± 1358 U/mL, Table 1) vs. BOL (21 ± 12 U/mL, *P*-value = 1.4 × 10^−7^, Table 1). Likewise, HE4 levels were higher in HGSOC (539 ± 1185 pmol/L, Table 1) vs. BOL (66 ± 25 pmol/L, *P*-value = 2.2 × 10^−7^, Table 1). Also expected was the strong correlation between CA125 and HE4 in the entire cohort and HGSOC (Fig. 2C and B). Malignancy was strongly correlated with both CA125 (rho = 0.63, *P* < 0.0001, Fig. 2C) and HE4 (rho = 0.67, *P* < 0.0001, Fig. 2C) before adjusting for age as well as after (Supplemental Fig. S2C).

## Discussion

### Cholesterol and lipids are essential to ovarian carcinoma

It is increasingly recognized that cholesterol plays a role in cancer progression [4]. Lipids are essential to rapidly dividing cells, especially tumor cells [12, 23–25]. OCs are no exception: they require lipids and cholesterol for their growth [12, 23, 24]. Alterations in the various pathways (intracellular synthesis vs. dietary vs endogenous pathways) through which OCs acquire their lipids still need to be clarified [26]. The objective of the present study was to measure circulating levels of Lp(a), Apo CIII, ANGPTL3 and PCSK9 in women diagnosed either with a high-grade serous ovarian carcinoma (HGSOC) or a benign ovarian lesion (BOL), given the availability of monoclonal antibodies and antisense therapy targeting these lipid-related factors.

### ANGPTL3 levels increase in HGSOC

This study was conducted to investigate the impact of malignant ovarian tumors on lipid-related factors. The study found that ANGPTL3, a well-established proangiogenic and hyperlipidemic factor, was higher in women diagnosed with HGSOC (BOL: 67 ng/mL, HGSOC: 84 ng/mL, Table 1). However, this 25% increase in ANGPTL3 levels did not result in any change in the lipid profile. Presence of HGSOC disrupted the association between ANGPTL3 and the lipid profile (see Fig. 2). It was observed that the lipid profile was similar between women with BOL and HGSOC (Table 1). It is important to note that ANGPTL3 origin (liver vs HGSOC) cannot be identified through measurements from peripheral blood venipunctures. Yet, higher expression of ANGPTL3 was reported in biopsy-confirmed HGSOC tissues by Siamakpour-Reihani (discussed below), supporting a contribution of an HGSOC-derived ANGPTL3 secretion in addition to the liver ANGPTL3 secretion.

### Lipoproteins and cholesterol levels in HGSOC

Lipids and their metabolic pathways have been linked to OCs [2, 27–30]. To establish whether malignant ovarian tumor influence circulating lipids in patients, the lipid profiles of women with BOL and HGSOC were compared. No significant change in TC, triglycerides, HDL, LDL, non-HDL, Apo B nor Lp(a) levels was observed between groups. These results concur with a study from 2007 reporting no difference in TC, LDL or HDL serum levels between 30 healthy controls and 32 patients of similar age with breast or ovarian cancers [31]. They contrast with a recent meta-analysis of 12 different studies reporting a decrease in TC and HDL in patients with ovarian tumors [32]. This discrepancy may be explained by the time separating blood sampling from diagnosis. A recent analysis of prospective case-control studies showed a possible inverse association between OC risk and TC levels when measured at least 2 years before diagnosis [33]. Furthermore, previous experimental and observational studies have proposed that levels of oxidized LDL, rather than total LDL, tend to show a stronger association with tumorigenesis and metastasis in OCs [31, 34, 35]. In the present study, oxidized LDL levels were not measured.

Another interesting observation was the lack of correlation between TG and HDL cholesterol levels in HGSOC patients (Fig. 2A vs. 2B for correlations; Supplemental Figs. S2A vs S2B for partial correlations with age-adjustment). In women without cancer, the expected strong and inverse correlation between HDL cholesterol and TG was present (rho = − 0.70, *P*-value < 0.000 1, *n* = 31, Fig. 2A). Meanwhile in women with OCs, no correlation was obtained between HDL cholesterol and TG (rho = − 0.25, non-significant *P*-value, *n* = 22, Fig. 2B). The latter observation goes against the well-established pathways linking HDL and TG. Nascent HDL emerge from the liver and acquire cholesterol from peripheral cells. Cholesteryl ester transfer protein (CETP) thereafter exchanges cholesterol esters from HDL to beta-lipoproteins for TG. As HDLs are enriched in TG, HDL cholesterol decreases, which explains the negative correlation commonly observed between HDL cholesterol and TG [36, 37]. A decoupling of lipoproteins metabolic pathways is portrayed by the disappearance of this correlation in women with HGSOC, perhaps involving ANGPTL3 or CETP.

### ANGPTL3 and circulating lipids in HGSOC

ANGTPL3 inhibits both hepatic and extra-hepatic lipoprotein lipases. Since lipases are responsible for the hydrolysis of triglycerides, levels of triglycerides-rich lipoproteins such as very-low-density lipoproteins (VLDL), intermediate-density lipoproteins (IDL) and LDL are increased upon ANGPTL3-mediated lipase inhibition [38], contributing to higher circulating levels of lipids.

Changes in ANGPTL3 in OC were previously reported by Siamakpour-Reihani et al [39]: RNA microarray analyses performed on ovarian tissue specimens from 51 chemotherapy-naïve patients with advanced HGSOC revealed that higher expression of ANGPTL3 was associated with shorter survival. The results of higher circulating levels of ANGPTL3 in HGSOC compared to BOL (Table 1, Fig. 1) are, therefore, a finding that is in line with results described by Siamakpour-Reihani et al., but only to a certain extent, as survival data for HGSOC patients recruited between 2017 and 2020 are currently unavailable. Elevated levels of ANGPTL3 were not coupled to an elevation of HDL levels in HGSOC (Fig. 2B and Supplemental Fig. S2B). This observation is unexpected in light of known pathways governing the relationships between ANGPTL3, HDL and TG [40, 41]. Correlations between ANGPTL3 and other components of the lipid panel, namely TC and non-HDL (Fig. 2A, rho = 0.45, *P*-value < 0.01 and rho =0.36, *P*-value < 0.05 respectively), also weakened in the HGSOC group (Fig. 2B, rho = 0.32 and rho = 0.25 respectively, all *P*-values non-significant). These observations evoke a rupture in the interrelationship between ANGPTL3 and cholesterolemia in HGSOC.

### Possible implications of ANGPTL3 increase in HGSOC

Lipid profile similarity between BOL and HGSOC (Table 1), despite increased ANGPTL3 levels in HGSOC (Fig. 1), was compatible with the absence of correlation between lipids and ANGPTL3 in HGSOC (Fig. 2B). This implies that presence of OC disrupts the relationship between ANGPTL3 and lipid levels in this cohort. One explanation could be that circulating lipids are taken up by tumor cells, inducing an upregulation of the hepatic secretion of ANGPTL3 to maintain a preset cholesterolemia. Alternatively, increased levels of ANGPTL3 in HGSOC could reflect local production by the tumor itself [39]. In such context, ANGPTL3 may be secreted in the tumor microenvironment to mediate pro-angiogenic and pro-metastatic functions, as noted in other cancers [42, 43]. But the latter does not exclude other possible roles played by ANGPTL3, such as in lipolysis. The decoupling between circulating ANGPTL3 and lipid levels in OCs as well as the origin of ANGPTL3 increased secretion (tumoral vs hepatic) need to be further investigated.

### Study limitations and strengths

The present work is a cross-sectional study and therefore precludes any causal association between higher ANGPTL3 levels and OC. Nonetheless, the analyses showed an increase of ANGPTL3 levels in women with HGSOC compared to women with BOL, a finding in line with previous data reported by Siamakpour-Reihani et al. Circulating atherogenic particles showed strong correlative association with each other (Apo B, LDL, non-HDL), providing an internal validation for all steps conducted (from measurements of lipid-related blood parameters to statistical analyses). In that regard, correlations between ANGPTL3 and circulating lipids in the control group is a finding that will likely be observed in a larger cohort of women [44] (Fig. 2A).

Plasma samples came from non-fasting participants, which may elevate TG levels and introduce bias in LDL values calculated with the Friedewald equation. Despite the use of non-fasting patients’ samples, measured TG levels were < 5 mmol/L [45], which means that calculated LDL levels were not biased by participants’ fasting/non-fasting status. The missing information included participants’ ethnicity, hormone-replacement therapy use [46] and cholesterol-lowering drugs [47], factors that could have affected the lipid profile and lipid-related factors.

Controls corresponded to women presenting a non-cancerous ovarian lesion. These lesions (fibromas and benign cysts) have not been associated with variations in lipid profile. Therefore, women diagnosed with BOL should not have lipid profiles different from lipid profiles of healthy women.

Women did not differ in age and had similar lipid profiles in the control vs. case groups (Table 1). Despite the absence of intergroup differences, age was considered as a potential biological confounder in analyses (see Table 1 for paired comparison, Supplemental Fig. S2 for partial correlation analyses).

The limited number of individuals belonging to tumor stages I, II and IV (Table 1, *n* = 3, n = 3 and *n* = 4, respectively) resulted in unequal group sizes and lack of statistical power in group comparisons, which did not permit to study ANGPTL3 and other analytes changes through the different stages. The observed low number of samples belonging to women in stage I and II is in line with the fact that HGSOC is frequently diagnosed at an advanced disease stage, contributing to the high lethality of HGSOC. A small number of participants increases the risk of dismissing real differences due to lower statistical power. Nevertheless, analyses on small groups represent a cost-effective step for identifying parameters worth further investigation, such as ANGPTL3 in OCs. The fact that well-established associations are observed in this cohort of 71 women (ex; the strong correlation between atherogenic measures LDL, Apo B and Non-HDL) suggests that the increase in ANGPTL3 in HGSOC is a real phenomenon which needs to be further investigated. Significant results with narrow confidence intervals generally have good predictive value for reproducibility in larger groups [48].

## Conclusions

The main finding from this study was an elevation of plasma ANGPTL3 levels in women diagnosed with epithelial high-grade serous ovarian carcinoma, along with a decoupling between ANGPTL3 levels and the lipid profile in HGSOC. Given the availability of a monoclonal antibody against ANGPTL3 already used in patients with familial hypercholesterolemia, these results warrant further investigation of whether ANGPTL3 inhibition has therapeutic potential in ovarian cancers. Confirmation of ANGPTL3 inhibition as a therapeutical target will permit rapid repositioning of Evinacumab in OC.

### Supplementary Information

**Additional file 1: Fig. S1.** Flow chart of analyses performed on the study population. **Fig. S2.** Pairwise Spearman’s partial correlation analyses between all variables, with age-adjustment. Partial correlations based on age-adjustment are presented for **(A)** benign ovarian lesions (BOL), **(B)** high-grade serous ovarian carcinoma (HGSOC) and **(C)** the entire cohort (overall). Correlation coefficients (rho) are displayed in each matrix lower half-panel with color hues indicative of relationship strength (see rho coefficient scale). Correlation significance is expressed in upper half-panel (ns: non-significant, *: *P* < 0.05; **: *P* < 0.01; ***: *P* < 0.001; ****: P < 0.000 1). **Fig. S3.** CA125 and HE4 plasma levels between BOL and HGSOC. Significant level increase of tumor markers CA125 (BOL: *n* = 40, HGSOC: *n* = 31, *P* = 1.4E-7) and HE4 (BOL: *n* = 38, HGSOC: *n* = 23, *P* = 2.2E-7) was found in HGSOC compared to BOL. ****: *P* < 0.000 1.

## Data Availability

Datasets generated for this study are not publicly available for the sake of participants’ privacy and confidentiality surrounding current ancillary studies underway but may be made available by the corresponding author upon reasonable request.

## Abbreviations

ANGPTL3: Angiopoietin protein-like 3
Apo B: Apolipoprotein B
Apo CIII: Apolipoprotein CIII
BOL: Benign ovarian lesion
HDL: High-density lipoprotein cholesterol
HGSOC: High-grade serous ovarian cancer
IDL: Intermediate density lipoprotein
LDL: Low-density lipoprotein cholesterol
Lp(a): Lipoprotein a
mab: Monoclonal antibody
OC: Ovarian cancer
PCSK9: Proprotein convertase subtilisin/kexin type 9
TC: Total cholesterol
TG: Triglycerides
VLDL: Very low-density lipoprotein
y.o.: Years old
