# Supplementary material for: Pre-operative levels of angiopoietin protein-like 3 (ANGPTL3) in women diagnosed with high-grade serous carcinoma of the ovary
Source: Lipids Health Dis. 2024 Feb 27;23:59. doi: 10.1186/s12944-024-02038-8 (PMC10898078; doi:10.1186/s12944-024-02038-8)
Supplement: Supplementary file 1 — Additional file 1: Fig. S1. Flow chart of analyses performed on the study population. Fig. S2. Pairwise Spearman’s partial correlation analyses between all variables, with age-adjustment. Partial correlations based on age-adjustment are presented for (A) benign ovarian lesions (BOL), (B) high-grade serous ovarian carcinoma (HGSOC) and (C) the entire cohort (overall). Correlation coefficients (rho) are displayed in each matrix lower half-panel with color hues indicative of relationship strength (see rho coefficient scale). Correlation significance is expressed in upper half-panel (ns: non-significant, *: P < 0.05; **: P < 0.01; ***: P < 0.001; ****: P < 0.000 1). Fig. S3. CA125 and HE4 plasma levels between BOL and HGSOC. Significant level increase of tumor markers CA125 (BOL: n = 40, HGSOC: n = 31, P = 1.4E-7) and HE4 (BOL: n = 38, HGSOC: n = 23, P = 2.2E-7) was found in HGSOC compared to BOL. ****: P < 0.000 1. [file 12944_2024_2038_MOESM1_ESM.docx]

**Supplemental figures for manuscript “Pre-operative levels of Angiopoietin Protein-Like 3 (ANGPTL3) in women diagnosed with high-grade serous carcinoma of the ovary”**

Emilie Wong Chong, France-Hélène Joncas, Pierre Douville, Dimcho Bachvarov, Caroline Diorio, Frédéric Calon, Ann-Charlotte Bergeron, Jonatan Blais, Shuk On Annie Leung, Nabil Georges Seidah, Anne Gangloff

|  | |
| --- | --- |
| **Figure S1.** Flow chart of analyses performed on the study population. | |

| **A** |  | **Figure S2.** **Pairwise Spearman’s partial correlation analyses between all variables, with age-adjustment.** Partial correlations based on age-adjustment are presented for **(A)** benign ovarian lesions (BOL), **(B)** high-grade serous ovarian carcinoma (HGSOC) and **(C)** the entire cohort (overall).  Correlation coefficients (rho) are displayed in each matrix lower half-panel with color hues indicative of relationship strength (see rho coefficient scale). Correlation significance is expressed in upper half-panel (ns: non-significant, *: *P* < 0.05; **: *P* < 0.01; ***: *P* < 0.001; *****:* *P* < 0.000 1). |
| --- | --- | --- |
| **BOL** |  |  |
| **B** |  |  |
| **HGSOC** |  |  |
| **C** | ****  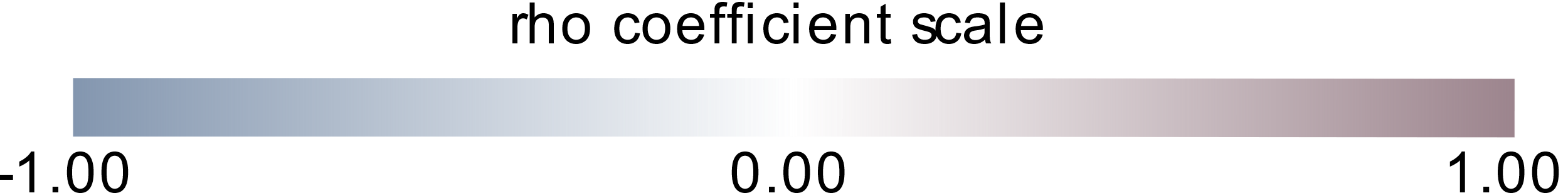 |  |
| **Overall** |  |  |

| 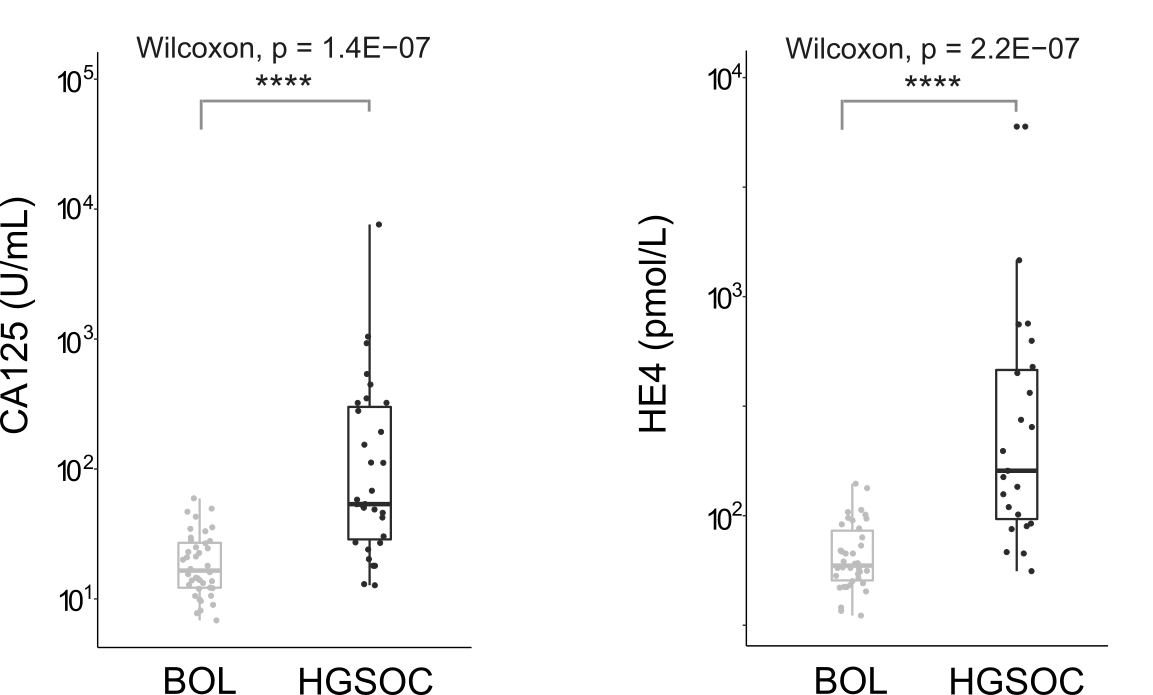 |
| --- |
| **Figure S3. CA125 and HE4 plasma levels between BOL and HGSOC.** Significant level increase of tumor markers CA125 (BOL: n=40, HGSOC: n=31, *P*=1.4E-7) and HE4 (BOL: n=38, HGSOC: n=23, *P*=2.2E-7) was found in HGSOC compared to BOL. ****: *P* < 0.000 1. |
